# Acidic pH-Induced Conformations and LAMP1 Binding of the Lassa Virus Glycoprotein Spike

**DOI:** 10.1371/journal.ppat.1005418

**Published:** 2016-02-05

**Authors:** Sai Li, Zhaoyang Sun, Rhys Pryce, Marie-Laure Parsy, Sarah K. Fehling, Katrin Schlie, C. Alistair Siebert, Wolfgang Garten, Thomas A. Bowden, Thomas Strecker, Juha T. Huiskonen

**Affiliations:** 1 Division of Structural Biology, Wellcome Trust Centre for Human Genetics, University of Oxford, Oxford, United Kingdom; 2 Institute of Virology, Philipps Universität Marburg, Marburg, Germany; National Institute of Health, UNITED STATES

## Abstract

Lassa virus is an enveloped, bi-segmented RNA virus and the most prevalent and fatal of all Old World arenaviruses. Virus entry into the host cell is mediated by a tripartite surface spike complex, which is composed of two viral glycoprotein subunits, GP1 and GP2, and the stable signal peptide. Of these, GP1 binds to cellular receptors and GP2 catalyzes fusion between the viral envelope and the host cell membrane during endocytosis. The molecular structure of the spike and conformational rearrangements induced by low pH, prior to fusion, remain poorly understood. Here, we analyzed the three-dimensional ultrastructure of Lassa virus using electron cryotomography. Sub-tomogram averaging yielded a structure of the glycoprotein spike at 14-Å resolution. The spikes are trimeric, cover the virion envelope, and connect to the underlying matrix. Structural changes to the spike, following acidification, support a viral entry mechanism dependent on binding to the lysosome-resident receptor LAMP1 and further dissociation of the membrane-distal GP1 subunits.

## Author Summary

Lassa virus is a zoonotic, hemorrhagic fever-causing pathogen. Because the virus can spread as an aerosol and there are no approved vaccines or specific antiviral drugs currently available, it poses a major impact on human health, affecting annually up to half a million people in West-African countries. Entry of the virus into the cells of the infected individual is the first step where the virus could be stopped. When we try to determine the molecular mechanism of virus entry, information on the structure of the virus and its components can be highly valuable. However, in the case of Lassa virus, this remains poorly understood. Here, we used high-resolution electron cryomicroscopy and tomography techniques to image chemically-inactivated Lassa virus. Computational image reconstruction allowed determination of the three-dimensional structure of the virus, and allowed us to localize the protein, lipid bilayer, and RNA genome components. Our analysis of non-infectious virus-like particles at different pH’s and in the presence of a functional cellular receptor, human LAMP-1, revealed specific structural rearrangements in the surface protein spike, providing a molecular-level rationale for this important stage of host cell entry.

## Introduction

Lassa virus (LASV) is an enveloped, ambi-sense, bi-segmented RNA virus endemic throughout Western Africa and is the most lethal of all known Old World arenaviruses. Due to the high mortality rates amongst hospitalized patients (~15%), ability of the virus to be spread by aerosol, and absence of licensed protective vaccines or therapeutics to treat acute infection, LASV has been classified as a biosafety level (BSL) 4 pathogen [1].

The LASV RNA genome encodes an RNA-dependent RNA polymerase (L), nucleoprotein (NP), matrix protein (Z), and a highly-glycosylated membrane glycoprotein (GP). GP is synthesized as an inactive precursor preGPC, which is co-translationally cleaved by signal peptidase into GPC and the stable signal peptide (SSP) [2]. Post-translational maturation cleavage of GPC by host protease SKI-1/S1P yields the receptor-binding subunit GP1 and the membrane-spanning fusion subunit GP2 [3–5]. SSP is not only critical by functioning as a *trans-*acting maturation factor [6], but also associates with the GP2 subunit, resulting in a tripartite mature GP complex on the viral surface [7,8].

Previous chemical cross-linking studies and sucrose density gradient analysis have revealed that GP complexes assemble on the virion surface as trimeric spikes [9]. Crystallographic analysis of GP1 has recently identified a putative binding surface for the lysosome-associated membrane protein 1 (LAMP1) [10], an essential intracellular receptor [11]. Structural studies on glycoproteins from related arenaviruses have further revealed that the GP1 forms a novel α/β fold [12,13] and that the GP2 is a class I fusion protein [3,5,14]. However, no three-dimensional (3D) structures exists to date for the whole LASV spike and there is thus paucity in our understanding the molecular architecture of LASV which in turn is important for understanding the mechanism of LASV entry.

Here, we have studied the ultrastructure of fixed Lassa virions by using electron cryomicroscopy and tomography. By averaging 6,500 spike densities, we derived the structure of the full-length GP spike in its pre-fusion conformation at 14 Å resolution. At this resolution, the trimeric arrangement and contacts to the underlying matrix were resolved. Low pH structures of GP trimers, derived from virus-like particles (VLPs), allowed assessing the conformational changes upon acidification. Addition of purified LAMP1 fragment allowed mapping its binding site on the spike and fitting of the GP1 X-ray structure [10] to the EM density allowed determination of its putative membrane-distal location. At pH below 5.0, the GP1 subunit was shed, possibly priming the GP2 for fusion. Taken together, these results help to map the architecture of Lassa virions and shed light on the conformational changes and internal receptor binding occurring during endosomal entry.

## Results

### Ultrastructure of Lassa virus

We produced live LASV in African green monkey kidney epithelial cells under BSL-4 containment conditions. Cell supernatants containing LASV virions were subsequently inactivated by chemical cross-linking using 4% paraformaldehyde (PFA). Prior to structural analysis, we optimized a gradient ultracentrifugation based protocol for virion purification (see Materials and Methods).

**Fig 1:**
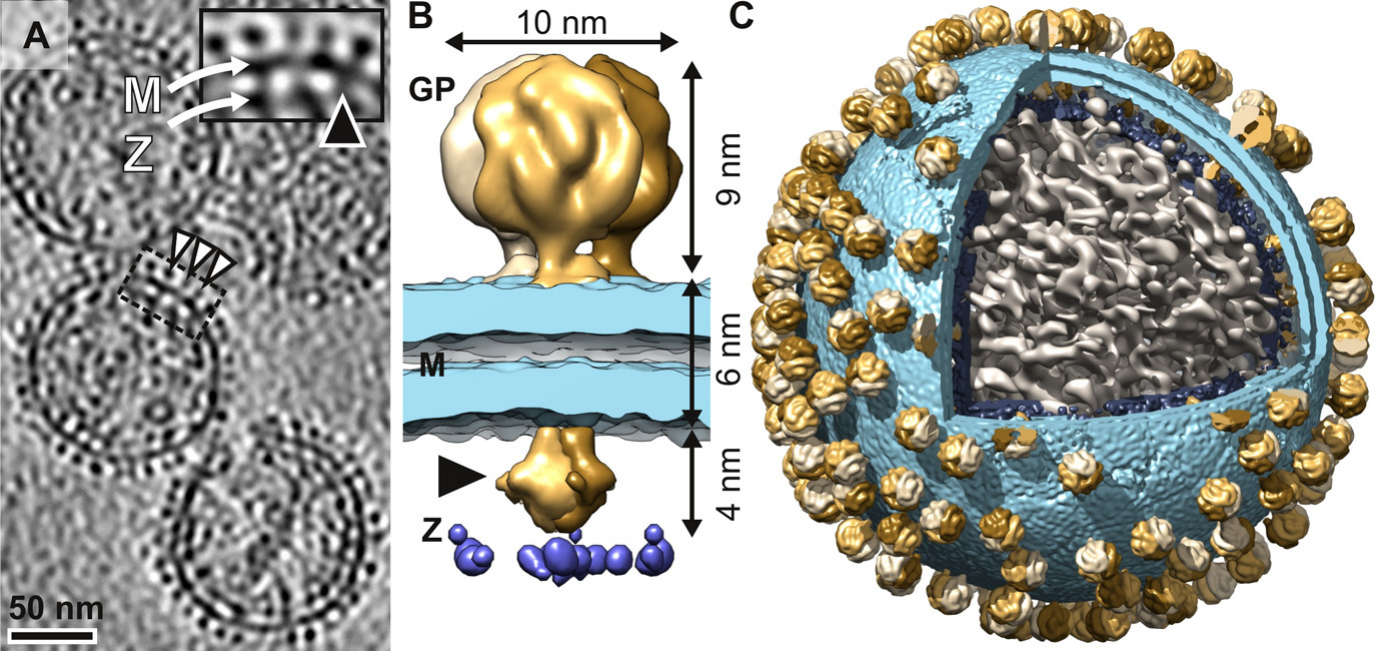
Structure of Lassa virus solved using electron cryotomography and sub-tomogram averaging. (A) A slice through a 3D tomogram of LASV. Three glycoprotein are spikes indicated (white arrowheads). The inset shows a magnified view of the area indicated (dotted line). The membrane (M), matrix (Z), and density bridging a spike to the Z layer (black arrowhead) are indicated. (B) LASV GP spike structure segmented to three spike protomers (different shades of brown), membrane (light blue), and the Z layer (dark blue). Intra-viral tails connecting the spike to the underlying matrix are indicated (arrowhead). (C) One LASV virion is shown cut open highlighting the irregular organization of the glycoprotein spikes, membrane, Z layer, and RNP density (gray).

Tomographic 3D reconstructions (‘tomograms’) calculated from electron cryomicroscopy tilt series of purified virions revealed both the exterior and interior density of LASV virions (Fig 1A). The virions were roughly spherical (S1 Fig, LASV) with diameter of 132±22 nm (S2 Fig, LASV). Consistent with previous observations [15], a density plot calculated normal to the membrane (S3 Fig, LASV) revealed i. an outer layer, comprising the GP spikes, ii. two middle layers, comprising the two leaflets of the lipid bilayer, and iii. two internal layers (‘inner track 1’ and ‘inner track 2’) [15], the first comprising GP intra-viral tails (see below) and the second comprising Z and possibly ribonucleoprotein (RNP), which also partially fills the virion interior (Fig 1A).

### Glycoprotein spike trimers connect to the underlying matrix and are randomly distributed on the virion surface

By averaging ~6,500 volumes (‘sub-tomograms’) of GP spikes extracted from tomograms of 48 inactivated LASV virions, we solved the 3D structure of the GP spike (Fig 1B and 1C and Table 1) to 14 Å resolution, as estimated by the Fourier shell correlation (FSC; S4 Fig). The structure revealed a trimeric assembly, measuring ~9 nm in height and ~10 nm in width (Fig 1B). Three separate densities (‘legs’), spaced by ~4 nm, anchor the spike to the membrane surface. By analogy to the trimeric structures of the HIV-1 [16] and Ebola virus glycoprotein [17] spikes, where the class I fusion glycoproteins subunits are membrane-proximal, we putatively assign these membrane proximal legs to the GP2 glycoprotein. This is supported by the presence of a trans-membrane (TM) region at the C-terminus of the LASV GP2, as predicted by sequence analysis. As SSP interacts with GP2 [8,18], it may also contribute to these leg densities. Earlier biochemical studies have suggested several topologies for SSP [8,19–21]. However, at 14-Å resolution we were unable to resolve the structure of SSP, making structural arrangements and orientation of SSP within the membrane proximal region difficult to predict. Furthermore, at this resolution the TM regions cannot be resolved and were thus not visible in our structure.

**Table 1:** Data acquisition and processing statistics.

	LASV	VLP	VLP	VLP+LAMP1	VLP
		pH 7.3	pH 5.2	pH 5.5	pH 3.0
**Data acquisition**					
Magnification	37,037	37,037	37,037	37,037	37,037
Tilt series	27	31	16	30	10
Tilt range (°)	-45–45	-45–45	-45–45	-45–45	-45–45
Interval (°)	5	5	5	5	5
Frames per tilt	8	8	8	8	8
Dose (e^–^/Å^2^)	~60	~60	~60	~60	~60
Defocus (um)^a^	2.3–3.9	1.7–3.7	2.8–6.7	1.4–4.6	3.0–3.9
**Data processing**					
Virions/VLPs	48	112	22	113	23
Seeds ^b^	n/a	n/a	9,406	n/a	7,821
Sub-tomograms	6,496	2,764	2,578	8,527	1,454
Box size (pixels)	128	128	128	128	128
Pixel size (Å)	2.7	2.7	2.7	2.7	2.7
Resolution (Å) ^c^	13.6	13.9	16.4	14.8	16.7

^a^Positive defocus denotes underfocus.

^b^Number of ‘seeds’ created on the virion/VLP surfaces. If n/a is indicated, manual picking was used instead of seeds.

^c^Resolution (Fourier shell correlation = 0.5).

Visual inspection of the tomographic slices revealed connections bridging the spike to the Z layer underneath the membrane (Fig 1A, inset). Density corresponding to these connections was ordered in the averaged GP structure (Fig 1B). The measured volume of this intra-viral density corresponded to molecular mass of ~18 kDa. This mass is consistent with the three 41-residue long intra-viral tails of GP2 (14 kDa in total), indicated by sequence analysis. We thus putatively assign the 18-kDa intra-viral density collectively to the three tails of GP2. In addition, SSP may contribute additional mass to these tails.

Template matching allowed mapping the distribution of spikes on the virions (N = 48). The spikes (273±22) almost fully covered the virion surface (Fig 1C). No higher-order clustering was observed (S5 Fig), confirming the well-accepted notion that LASV is not icosahedrally symmetric and suggesting that no specific interactions between the GP trimers exist.

### Structure of the native LASV GP from virus-like particles

To account for possible effects of chemical fixation, we exploited a previously reported stable cell line expressing non-infectious virus-like particles (VLPs) displaying the full-length LASV GP but lacking the Z [22]. Similar to the fixed virions, VLPs purified from cell culture supernatants were covered with GP spikes (S1 Fig; VLP pH 7). However, size measurements (S2 Fig) showed that VLPs were significantly smaller (diameter 82±15 nm) than the virions (132±22 nm) and also their shape was more variable (S1 Fig). This is most likely due to the absence of Z, which modulates virion budding and RNP packaging [23].

**Fig 2:**
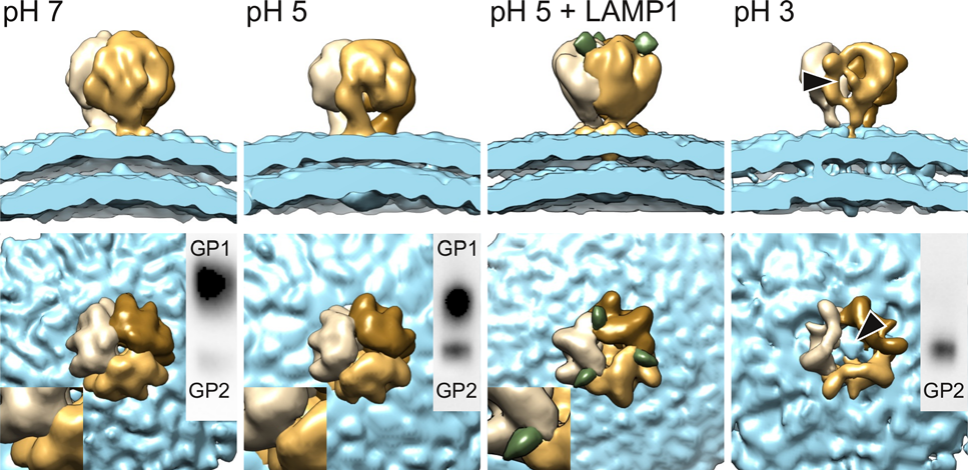
Structural transitions and LAMP1 binding of the LASV GP spikes upon acidification. (A) GP structures at different pHs are shown from side (top row) and top (bottom row). All volumes were filtered to 17-Å resolution, rendered at molecular threshold corresponding to the expected molecular mass, and colored as in Fig 1. Residual density corresponding to LAMP1 is colored in green. Inserts in the lower left corners show a close-up of the interface between two spike monomers. Insets in the top right corners show Western blot analysis of GP1 and GP2 subunits. The arrowheads indicate the missing density in the central top part and side of the pH 3 structure.

Following the same sub-tomogram averaging approach as for the virions, we solved the structure of the native GP spike from the VLPs (Fig 2, pH 7; Table 1) to 14-Å resolution (S4 Fig). Visual inspection revealed that the VLP-derived spike ectodomain structure shared the same tripodal architecture and structural features with the virion-derived spike structure (S6A Fig). To provide a quantitative measure for similarity, we performed cross-FSC analysis, which indicated that the two ectodomains agreed to 16-Å resolution (S6B Fig).

The density profile calculated across the VLP membrane was practically identical to that of the virion for the density corresponding both to the GP ectodomain and GP2 intra-viral tails. As expected, density assigned to Z in the virion was absent in the VLPs that lacked this component (S3 Fig, VLP). Surprisingly, the GP2 intra-viral tails that were ordered in the virions (Fig 1B) were disordered in the VLP-derived GP structure (S6A Fig). While it is possible to attribute the ordering of the intra-viral tails to the GP–Z interaction [22,24], we cannot exclude the possible effect of chemical fixation in ordering the intra-viral tails.

The direct comparison of the virion-derived and VLP-derived GP structures supports our assignment of the density in the first density layer under the membrane (denoted earlier as ‘inner track 1’) [15] to the GP2 intra-viral tails. We note that this assignment differs from a previous assignment, where the ‘inner track 1’ was assigned to the Z layer in the two-dimensional cryo-EM images of three other arenaviruses, namely Pichinde, Tacaribe and lymphocytic choriomeningitis virus [15]. We note, however, that we cannot exclude structural differences between LASV and the other studied arenaviruses. It is also possible that Z contributes some density to the inner track 1 in LASV. In conclusion, by comparison to the VLP system, we could verify the LASV GP spike structure and our assignment of the intra-viral GP2 density.

### Glycoprotein spike binding to LAMP1 at endosomal pH

Following virus internalization into the host cell through GP attachment to α-dystroglycan [25], DC-SIGN [26,27], or other cellular receptors [26], fusion of viral and host cell membranes occurs at an unusually low pH of 3–4.5 [28], a process mediated by recognition of the host lysosomal receptor, LAMP1 [11]. Receptor binding is modulated by pH; GP binds to α-dystroglycan at pH 8.0 but not at pH 6.0 and below, and, conversely, LAMP1 binding occurs only at pH 6.0 and below [11].

To study GP conformation at endosomal pH, we determined the VLP-derived GP structure at pH 5.2 (Table 1 and Fig 2, pH 5, and S1 Fig VLP pH 5). The structure was resolved at 16-Å resolution (S4 Fig) and revealed conformational differences when compared to the same structure at pH 7.3 (Fig 2, pH 7). These can be attributed to a conformational difference at the membrane-proximal legs and a slight opening up of the spike, creating crevices at the top of the spike between the GP subunits.

To map the LAMP1 binding site on the GP spike, we repeated the electron cryomicroscopy-based structure analysis after addition of purified LAMP1 at pH 5.5 (Fig 2, pH 5 + LAMP1). First we expressed and purified the predicted membrane distal, highly glycosylated β-prism domain of human LAMP1 (residues 27–194) [29]. This domain was biologically active binding to VLPs at pH 5.0 (S7 Fig). The GP–LAMP1 complex structure was resolved to 15-Å resolution (S4 Fig). Additional density attributed to LAMP1 was evident at the top of the spike and located close to the crevices observed in the VLP GP structure in absence of LAMP1 (Fig 2; pH 5 + LAMP1). The volume of LAMP1 density was less than expected based on the molecular mass of the LAMP1 glycoprotein fragment used for analysis (S7 Fig), suggesting that LAMP1 either bound to VLP GP in sub-stoichiometric amounts and/or that the binding was flexible. In conclusion, the location of this additional residual density allowed mapping of the LAMP1 binding site to the membrane distal part of the spike.

### GP1 subunit is shed from the spike at lysosomal pH

In line with previous reports [11], our Western blot analysis revealed that the GP1 subunit was shed from the VLPs at pH 3.0 and 4.0 (S8 Fig). To determine the LASV GP structure in the absence of GP1, we performed electron cryotomography and sub-tomogram averaging of VLP-derived GPs at pH 3.0 (Table 1 and S1 Fig, VLP pH 3). The structure was missing density in the membrane-distal part of the spike (Fig 2, pH 3), suggesting that the GP1 occupies this region.

In the absence of a target membrane, the pH 3 structure may constitute SSP in complex with either a non-productive post-fusion conformation of GP2 or an intermediate conformation prior to fusion. Indeed, it is strikingly different from the canonical post-fusion ectodomain structures of other class I fusion proteins, which display a central trimeric alpha-helical coiled coil (S9 Fig) [3,30]. However, we also cannot exclude the possibility that a fraction of GP2 has mediated fusion of some VLPs with one another. The resulting post-fusion conformation species in this fraction may have been excluded from our analysis. Despite these limitations, the pH 3 structure allowed putative localization the GP1 subunit to the membrane distal region of the spike. This is consistent with the mapped LAMP1 binding site in the same region, as LAMP1 binds to the GP1 subunit [10].

### Fitting of GP1 structure into the GP spike density

**Fig 3:**
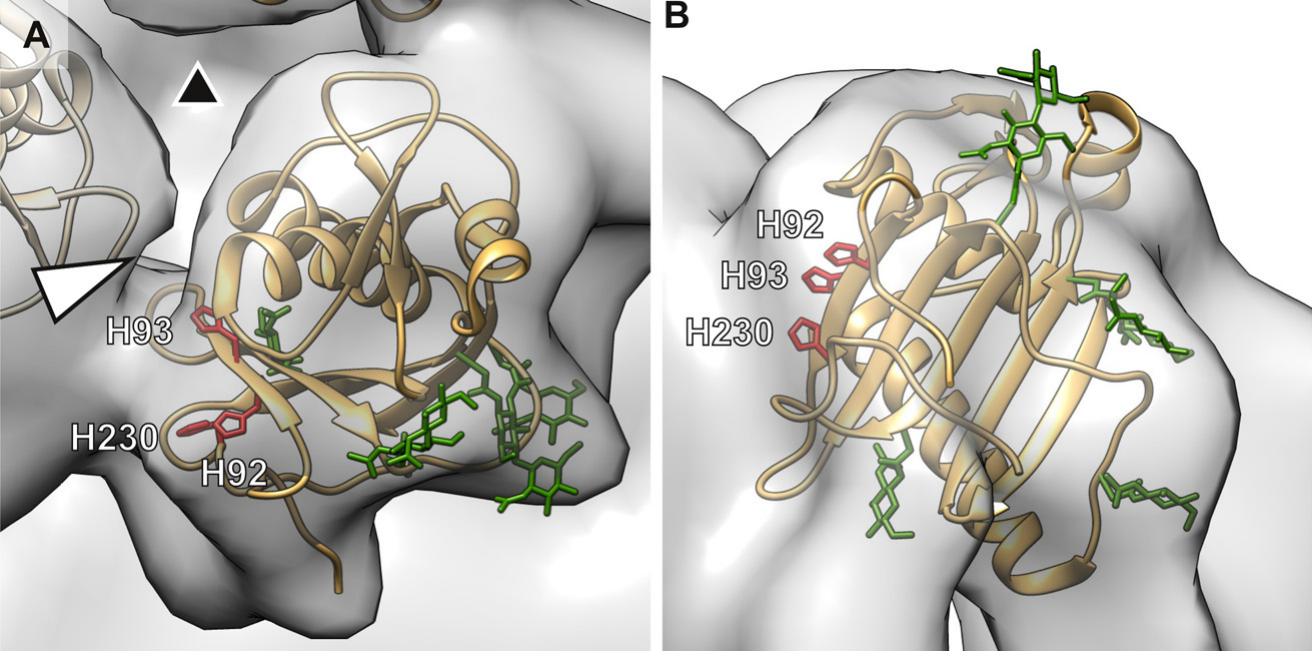
Location of the GP1 subunit in the glycoprotein spike. (A) Crystallographic structure [10](brown ribbon; PDB:4ZJF) was fitted into density (transparent surface) derived by electron cryomicroscopy and subtomogram averaging of GP spikes from virus-like particles at pH 5. The glycans are represented with sticks and colored in green. The histidines indicated in binding of an intracellular receptor (lysosome-associated membrane protein 1, LAMP1) are labeled and colored in red. This triad of histidines faces a crevice (white arrowhead) between the spike subunits. The three-fold symmetry axis is indicated with a black triangle. (B) Same fitting as in *A* is shown from the side.

The structure of LASV GP1 has been recently solved by X-ray crystallography [10]. To localize the GP1 and determine its orientation relative to the LAMP1 binding site, we used volumetric correlation to fit the GP1 crystal structure into our GP reconstruction (Fig 3 and S10 Fig). As the GP1 has been crystallized at pH 5 and acidic pH has been suggested to have an effect on GP1 conformation [10], we used our GP structure derived from VLPs at pH 5 for fitting. The fitting was unambiguous (S10C Fig; cross-correlation coefficient 0.86) and placed the GP1 structure to the membrane distal part of the spike (Fig 3). Interestingly, the triad of histidines that has been indicated in LAMP1 binding [10] points towards the crevice between GP1 subunits (Fig 3), the putative LAMP1 binding side (Fig 2). The histidines may act as a pH sensor [10] opening up the spike at pH 5 to create a binding site for LAMP1, but further studies are needed to test this hypothesis.

## Discussion

Viral surface glycoproteins mediate entry of enveloped viruses into host cells through receptor binding and subsequent fusion of viral and cellular membranes. Therefore, the structural characterization of viral glycoproteins is of fundamental interest to understand the molecular mechanisms underlying host cell infection. LASV uses a two-step process to enter target cells during infection. Upon receptor binding at the cell surface, LASV enters the cell through late endosomes/multivesicular bodies, where GP undergoes a pH-induced switch to engage the intracellular receptor LAMP1 [11,31].

We analyzed the three-dimensional ultrastructure of authentic LASV using electron cryotomography. Sub-tomogram averaging yielded a structure of the glycoprotein spike at 14-Å resolution. To our knowledge, the GP structures presented here constitute the highest resolution viral glycoprotein spike structures solved *in situ*. The structure revealed a trimeric glycoprotein architecture, which confirms previous chemical cross-linking studies, demonstrating the formation of trimeric mature glycoprotein spikes on the surface of LASV particles [9].

Our analysis of VLPs harboring GP revealed that the VLPs were significantly smaller in size (diameter 82±15 nm) than the virions (132±22 nm) and that their shape was more variable; possibly due to the absence of other viral components. Despite these morphological differences, comparison of the GP spike structures between virions and non-infectious VLPs showed that they share high degree of structural similarity. It is thus likely that the GP-harboring VLPs mimic at least to some extent the immunological properties of the authentic virions, which is critical for the use of VLP-based vaccine platforms for prophylactic protection against Lassa fever [32].

Although the outer spike domain on VLPs appeared structurally similar to LASV virions, differences were observed for the intra-viral domain architecture. The intra-viral GP2/SSP tails were ordered in LASV virions, however, in the VLP-derived GP structure they were disordered. The arenaviral matrix protein Z plays an important role in viral assembly and budding, acting as a bridging factor between the virus envelope spike complex and the viral RNP [23]. Thus, the assembly of infectious LASV involves an interaction between Z and the cytoplasmic portion of the GP complex, which is supported by our finding that Z is positioned beneath the viral bilayer and connected with the spike in LASV virions. Interestingly, arenaviruses are unique in that Z associates via its N-terminal myristylation with SSP, even in the absence of other subunits of the GP complex [24]. However, the detailed organization of SSP in the tripartite GP complex and the interactions between the GP complex and Z remain elusive. Furthermore, how Z potentially contributes to the stabilization of the GP intra-viral domain remains to be determined.

In addition to providing an independent control for our structural analysis, the VLP system allowed us to probe the structural states of the GP in acidic conditions, similar to those encountered by LASV during endocytotic entry. In another group of membraneous viruses, alphaviruses, the E1 fusion glycoprotein is shielded by E2 glycoprotein preventing premature fusion [33]. Here, the membrane-distal GP1 remained bound to the membrane-anchored GP2 at pH 5.0, possibly providing an analogous shielding function and at the same time creating a binding site for the intra-cellular receptor LAMP1.

**Fig 4:**
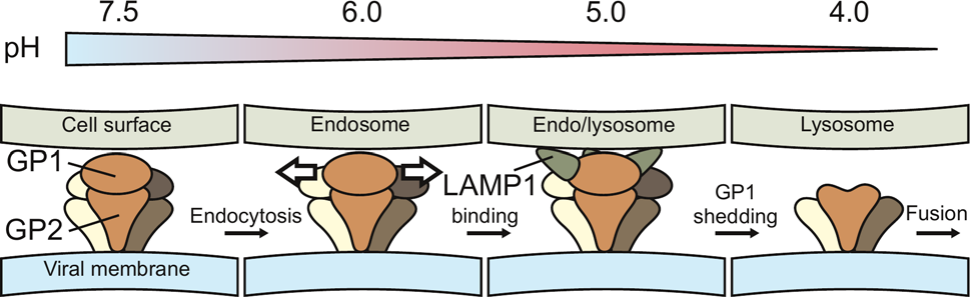
Model for Lassa virus entry. A schematic representation of a hypothetical entry model, derived from the structures determined in this study, is shown. An approximate range of pH in different cellular compartments is depicted as a color gradient from neutral (blue) to very acidic (red). Lassa virus glycoprotein (GP) spike trimer is depicted in three shades of brown and the two different subunits (GP1 and GP2) are labeled. Lysosome-associated membrane protein 1 (LAMP1), an intracellular Lassa virus receptor, is labeled and colored in green. Viral membrane is colored in blue. Different cellular membranes are labeled and colored in gray. Formation of the crevices between GP1 subunits is indicated with white arrows. See text for full description of the entry model.

To rationalize the findings from our structural analysis, we propose a hypothetical entry model (Fig 4). In this model, GP1 structure opens up at endosomal pH, creating a crevice between the membrane distal GP1 subunits and exposing a binding site for LAMP1. Interestingly, based on the GP1 crystal structure a pH dependent conformational change in GP1 has been suggested recently, preventing α-dystroglycan and enabling LAMP1 binding [10]. It is tempting to hypothesize that LAMP1 promotes detachment of GP1 from GP2 but the role of LAMP1 in promoting LASV fusion still remains unclear. We further suggest that detachment of GP1 at lysosomal pH primes the GP2/SSP complex for membrane-fusion [7,28,34]. Prior to membrane fusion, GP2 is expected to embed the hydrophobic fusion peptide into the target membrane [35] and further to adopt a post-fusion trimeric alpha-helical coiled coil characteristic of class I fusion proteins [3,30]. The organization of SSP during this process remains unknown.

**Fig 5:**
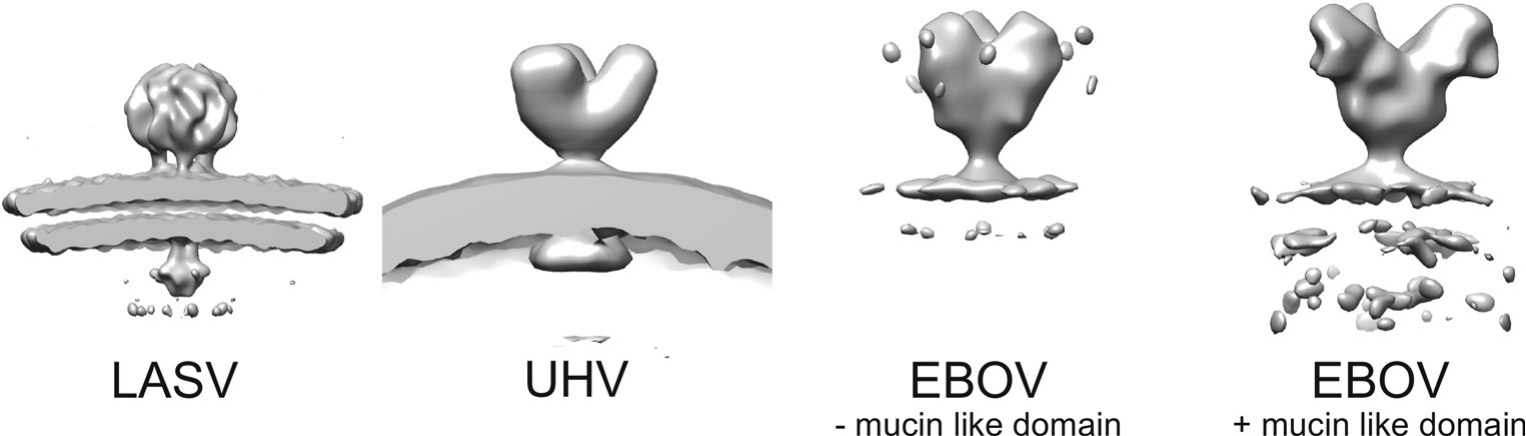
Comparison of arenavirus and filovirus glycoprotein spike structures. Structures of Lassa virus spike (LASV, this study), spike of the arenenavirus-like virus infecting snakes (University of Helsinki Virus, UHL; EMD-2424), and Ebola virus spike (EBOV) with (EMD-6003) and without (EMD-6004) the mucin-like domain are shown at the same scale for comparison.

Interestingly, the observed three-legged architecture of LASV GP differs from that reported for a novel arenavirus-like virus infecting snakes [36], belonging to the genus Reptarenavirus within the *Arenaviridae* family, which is surprisingly more similar to the glycoprotein spike of Ebola virus [17] (Fig 5). This observation may reflect both the structural diversity amongst mammalian and reptilian arenaviruses and an evolutionary link to filoviruses [37]. In conclusion, our structures provide a blueprint for the surface of mammalian arenaviruses and a model for the structural rearrangements that occur during host cell entry.

## Materials and Methods

### Preparation, inactivation and purification of LASV

All work with infectious LASV was performed under the highest safety precautions in the biosafety level-4 (BSL-4) facility at the Institute of Virology in Marburg. Vero cells were infected with LASV (strain Josiah) at a multiplicity of infection of 0.1. Infected cells were maintained in Dulbecco's modified Eagle medium (DMEM; Gibco, Thermo Fischer Scientific, Waltham, MA) supplemented with 2% fetal calf serum (FCS; Gibco, Thermo Fischer Scientific, Waltham, MA), penicillin (100 U/ml), streptomycin (100 mg/ml), and L-glutamine (2 mmol/l) (Invitrogen, Thermo Fischer Scientific, Waltham, MA) at 37°C under 5% CO_2_. Supernatant was collected 5 days after infection and cleared twice by centrifugation at 5,000 g for 30 min. Supernatant containing LASV was then subjected to virus inactivation using PFA (4% final concentration) in DMEM for 24 h. The sample was removed from the BSL-4 facility and after additional 24 h processed for further experiments.

Supernatants containing PFA-fixed LASV (30 ml) were again cleared twice by low-speed centrifugation (5,000 g for 30 min) to remove potential PFA-induced aggregates and subsequently pelleted through 20% sucrose cushion by ultracentrifugation (100,000 g for 2 h). Pellets were resuspended in 150 μl phosphate-buffered saline (PBS) for 12 h at 4°C. The samples were further purified by pelleting twice through 20% sucrose cushion by ultracentrifugation (100,000 g for 2 h) and resuspended in PBS. The sample was laid onto a 10%–60% sucrose gradient and centrifuged (100,000 g for 12 h). The gradient was fractionated using a gradient fractionator (Biocomp, Fredericton, NB, Canada) and fractions were collected using a fraction collector (Gilson, Middleton, WI) while monitoring the UV absorbance at 254 nm using a BioProbe (Biocomp, Fredericton, NB, Canada) together with EM-1 UV monitor (Bio-Rad Laboratories, Hercules, CA). Ten 100-μl fractions collected around the absorbance peak were pooled, pelleted and resuspended as described above.

### Expression and purification of virus-like particles

Madin–Darby canine kidney (MDCK-II) cells stably expressing LASV GP protein [22] were cultured in DMEM supplemented with 10% FCS for 96 h. The medium was replaced by DMEM with 2% FCS for VLP expression. After 96 h, the cell supernatant was cleared twice by centrifugation (5,000 g for 30 min). Virus-like particles (VLPs) were pelleted through 20% sucrose cushion by ultracentrifugation (125,000 g for 3 h). Pellets were resuspended in 150 μl PBS for 12 h at 4°C.

Solutions of succinic acid, sodium dihydrogen phosphate and glycine were mixed in 2:7:7 molar ratio to make 50 mM SPG buffer. The buffer was adjusted to the desired pH by adding 0.5 M sodium hydroxide or 1 M hydrogen chloride. For Western blot analysis of particles at different pH, 300 μl of sample was incubated with 5 ml SPG buffer at the desired pH for 5 min at room temperature, then pelleted through sucrose cushion and resuspended as described above. Protein concentration of purified and different pH treated VLPs was measured using Nanodrop (Thermo Fischer Scientific).

### Western blot analysis

An aliquot corresponding to 8 μg protein from each sample was separated by SDS-PAGE on a NuPAGE 4–12% Bis-Tris gel (Life Technologies, Thermo Fischer Scientific) and subsequently transferred to nitrocellulose membranes by using a dry-blotting system (iBlot; Life Technologies). Membranes were blocked in 5% milk in PBS buffer for 2 hours. Membranes were incubated with 1/3,000 diluted primary antibodies (monoclonal mouse antibody AC1 against GP-C and GP1 [kindly provided by Marie-Claude Georges-Courbot]; polyclonal rabbit antibody GP4 recognizing GP-C and GP2 [38]) for 1 h followed by an incubation with 1/1,000 diluted horseradish peroxidase- conjugated secondary antibodies against mouse and rabbit (Qiagen, Crawley, UK) for 1 h. The membrane was soaked in 5 ml of enhanced chemiluminescence reagent mixture (GE Healthcare, Buckinghamshire, UK) and visualized in a ChemiDoc MP gel imager (Bio-Rad Laboratories, Hercules, CA). A 1-μl aliquot of pre-stained protein maker (Benchmark; Life Technologies) was used to determine the protein mass.

### Expression and purification of human LAMP1 membrane distal domain

A fragment of human LAMP1 (residues 27–194; UniProt accession no. P11279), predicted to correspond to the membrane-distal β-prism domain [29], was cloned into the pHLsec mammalian expression vector [39]. The LAMP1 domain was transiently expressed in HEK293T cells in the presence of the α-mannosidase inhibitor, kifunensine [40], with 2 mg of DNA transfected per litre of cell culture. Cell culture supernatant was collected 3 days post-transfection, filtered and diafiltrated against 10 mM Tris (pH 8.0) and 150mM NaCl. Purification comprised a two-step immobilized metal-affinity and size-exclusion procedure. Size-exclusion purification was carried out using a Superdex 200 10/30 column equilibrated against 10 mM Tris (pH 8.0) and 150 mM NaCl.

### Binding of human LAMP1 membrane distal domain to VLPs

Enzyme-linked immunosorbent assays were performed in 96-well Nunc-Immuno PolySorp plates (Sigma) to which VLPs were bound overnight at a concentration of 10 μg/ml. Plates were blocked with PBS containing 5% milk powder and incubated with varying concentrations of purified LAMP1 membrane distal domain at different pH. LAMP1 detection was mediated using a rabbit derived anti-6His primary (Abcam, UK), and goat-anti rabbit-HRP (Abcam) antibody combination. Binding was assessed on the basis of optical absorbance at 405 nm after 30 min following HRP substrate addition.

### Cryo-EM sample preparation

An aliquot (3 μl) of purified LASV or VLP sample was mixed with 3 μl of colloidal 6-nm gold particles coupled to bovine serum albumin (Aurion, Wageningen, The Netherlands) and applied on a plasma cleaned EM grids coated with holey carbon (C-flat; Protochips, Raleigh, NC). For low pH treatment of VLPs, the grid was floated for three minutes on top of a 2-ml droplet of SPG buffer at the desired pH. The grids were vitrified using a plunger device (CP3; Gatan, Pleasanton, CA) by blotting the sample for 3 s followed by plunge-freezing into a mixture of liquid ethane (37%) and propane (63%) [41].

### Electron cryomicroscopy and tomography

All electron cryomicroscopy data were collected using a 300-kV transmission electron microscope (TF30 ‘Polara’; FEI, Eindhoven, Netherlands) operated at liquid nitrogen temperature. SerialEM [42] was used for acquiring low dose, single axis tilt series (from –45° to 45° with 5° angular sampling). A direct electron detector camera (K2 Summit; Gatan, Pleasanton, CA) mounted behind a post-column energy filter (QIF Quantum LS; Gatan, Pleasanton, CA) was used to acquire data at zero energy-loss mode (slit width 20 eV). At each tilt, a movie consisting of eight frames with total exposure time of 1.6 s was collected at a calibrated magnification of ×37,000 in electron counting super resolution mode, corresponding to a pixel size of 0.675 Å. Tilt series were recorded at nominal defocus values ranging from 2.0 to 3.5 μm under focus and the total electron dose was ~60 e^–^/Å^2^.

### Image pre-processing and tomogram generation

Drift correction [43] was used to correct for the electron beam induced motion by averaging eight frames for each tilt and applying 2× binning in Fourier space. IMOD package [44] was used to reconstruct three-dimensional tomograms from the stacks of tilted images. Images were aligned using the 6-nm gold beads as fiducial markers (S11 Fig). Prior to reconstruction, contribution from the gold beads was computationally removed, contrast transfer function (CTF) parameters were manually estimated (S12 Fig) and the effects of the CTF were corrected in the images by phase flipping [45]. For CTF background correction, noise images were recorded and processed the same was as actual tilt images. The amplitudes were not modified during CTF correction. Further 2× binning was applied, resulting in the final pixel size of 2.7 Å. LASV virions and VLPs were extracted from the full tomograms for further analysis.

### Sub-tomogram averaging

Sub-tomogram alignment and averaging was carried out in Dynamo, which takes into account the missing wedge in tomographic data and allows parallel and graphics processing unit accelerated computations [46]. For initial template generation, extracted virion and VLP volumes were low-pass filtered to 80 Å using Bsoft [47]. Spikes were manually picked from the volumes of LASV and VLPs at pH 7 and pH 5 using Dynamo [46]. Dynamo *tomoview* program was used to create a surface model approximating the viral membrane, assuming either spherical or ellipsoidal geometry. Surface normals of the model surface provided initial orientations of the picked spikes. Spikes were extracted from the unfiltered tomograms into boxes of 128×128×128 voxels and averaged together. Cylindrical symmetry and low-pass filter to 35-Å resolution were applied on the average to reduce template bias.

The orientations were refined using the standard refinement strategy in Dynamo that implements an adaptive low-pass (‘push-back’) filter to further mitigate effects of template bias. Iterative refinement was performed in several stages (Supplementary Methods in S1 Text) [48–50]. Initially, no symmetry was applied to not bias the structure towards any particular symmetry. Only after three-fold appearance became evident in the asymmetric average (S13A Fig; C1), three-fold symmetry was applied in subsequent iterations (S13A Fig; C3). We further tested the effects of the starting model on the resulting structure. Even when the refinement was started with an initial model with wrong symmetry, it converged to a trimeric structure (S13B Fig). Typically a total 20 iterations were run until the resolution stopped improving (S13C Fig). In addition to the standard ‘push-back’ refinement, we carried out further tests with an alternative, so called ‘gold-standard’ refinement strategy using the VLP pH 7 data set. Both strategies yielded a trimeric spike at comparable resolutions (S14 Fig; Supplementary Methods in S1 Text).

To increase the size of the VLP pH 5 dataset and to locate all spikes in the VLP pH 3 data, we used template matching restricted on the VLP membrane surface. First, evenly distributed and oriented pseudo-particles (denoted here as ‘seeds’) were created on the surface of the particles [49,50] using Dynamo *tomoview* function using an ellipsoidal or a spherical surface model [46]. Seeds were defined with a seed-to-seed spacing of 24 pixels, resulting on average 430 and 340 seeds for pH 5 and pH 3 VLPs, respectively. As the seeds can correspond both to membrane areas that have spikes (true positives) and to areas that are devoid of spikes (false positives), these need to be differentiated. We thus used two templates in a multi-reference refinement run. First, a low-pass filtered cylindrically averaged spike, generated from the earlier VLP pH 5 average, served as a template for the true positives. Second, a spike-free membrane model served as a template for the false positives. The template that correlated the best determined whether the sub-tomogram in the given ‘seed’ position was a true or false positive. Shifts of 20 pixels were allowed lateral to the membrane. Unique spike sub-tomograms were identified by removing seeds that were too close to another seed. The included sub-tomograms were then subjected to structure refinement similar to the manually picked spikes. No spikes selected for the refinement were excluded after this stage.

The positions of the seeds were then iteratively refined, similarly as described above for manually picked spikes. Low-pass filtered cylindrical average of the densities extracted at seed positions was used as an initial template. The seeds were allowed mainly to shift only in the direction normal to the membrane. A smoothness criterion was implemented as a custom Dynamo function to weight these shifts by the shifts of the neighboring seeds. Similarly to motion correction of particles in single particle processing [51], the weight of each neighbor was adjusted by Gaussian function taking into account the distance to the seed in question, the neighbors farther away from the seed contributing less than the neighbors closer to the seed.

The resolution of the final maps was estimated by FSC, by splitting the sub-tomograms into two half datasets and using 0.5 as threshold [52]. As sub-tomograms extracted from the same virion (or VLP) may not be fully independent, each virion (or VLP) contributed sub-tomograms to one of the half datasets, but not both.

### Structure analysis

To putatively assign densities to different structural components, the averaged density maps were computationally segmented using Segger [53] in UCSF Chimera [54]. The isosurface threshold in the spike averages was set to match the expected volume of the spike ectodomain (290,000 Å^3^), calculated from the estimated spike ectodomain trimer mass (230 kDa, including glycosylations) assuming density of 0.81 Da / Å^3^. For the spike at pH 3 lagging GP1, the volume was matched to correspond to three copies of GP2 (86 kDa; 106,000 Å^3^). Spike ectodomain densities were then segmented. The volume corresponding to the LASV GP intra-viral segments was measured in UCSF Chimera.

To create a composite model for the LASV virion, sub-tomogram refinement parameters were first converted from Dynamo tables to a STAR file compatible with Jsubtomo[50]. The segmented densities for the spike protomers, membrane and matrix layer were then placed on their original places relative to the virion by using *jsubtomo_create_model*.*py*. An isosurface representation, also showing the RNP density inside of the virion was rendered in UCSF Chimera.

### Fitting of atomic coordinates

We fitted the atomic coordinates of LASV GP1 (PDB:4ZJF) [10] to our VLP pH 5 spike using Segger [53]. Each segment assigned to a GP subunit was split into four smaller segments (S10A and S10B Fig). Each of the four chains present in the GP1 crystal structure were fitted to the four GP segments. From the total of 1,000 evenly rotated fits, unique fits were created by further optimization and by clustering fits that were less than 5.0 Å and 3.0 degrees apart (S10C Fig).

## Supporting Information

S1 Fig Electron cryotomography of Lassa virus (LASV) and virus-like particles (VLP) harboring GP. 8-nm thick slices through three-dimensional tomographic reconstructions of LASV and VLPs at different pH low-pass filtered to 60-Å resolution are shown. Scale bars, 50 nm.
(TIF)

S2 Fig Size distribution of LASV and virus-like particles (VLPs) at different pH. Particle size histograms are plotted for the LASV virion and VLPs at different pH. Mean diameter±standard deviation and the number of measurements (N) are indicated for each sample. The area of each fitted Gaussian distribution was normalized to 1.
(TIF)

S3 Fig Density distribution of LASV and virus like particle (VLP). Plots showing the average density distribution in LASV and VLP at pH 7. The insets show a slice through the averaged density, from which the plots were calculated. The glycoprotein layer (GP), membrane (M) and matrix layer (Z) are indicated. The GP intra-viral tails are marked with an asterisk. Notice that the peak assigned to the intra-viral tails is present in both LASV and VLP, but whereas the peak assigned to the Z-layer in present in LASV it is absent in VLP as expected. Both plots have been centered to the middle of the lipid bilayer.
(TIF)

S4 Fig Resolution estimation of the structures. Fourier shell correlation (FSC) is plotted for the LASV GP structure, in addition to the VLP structures at different pH and GP–LAMP1 complex. The resolutions at which the FSC drops below the threshold (0.5, dashed line) are indicated.
(TIF)

S5 Fig Glycoprotein spikes are distributed randomly on the virions. Histogram of pairwise distances of 13,060 spikes found by template-matching on 48 LASV virions is shown. Each bin of the histogram is 3 nm wide. The smallest pairwise distance (~10 nm) reflects the closest spike-to-spike packing corresponding to the size of the spike. The largest pairwise distance (~170 nm) reflects the size of the largest virions in the data set.
(TIF)

S6 Fig Comparison of glycoprotein spike structures from fixed LASV and native virus-like particles at pH 7. (A) An isosurface representation is shown for the GP structure derived from fixed virions (LASV) and from virus-like particles (VLP pH 7) from the side and from the top. (B) Fourier shell correlation (FSC) calculated between the spike ectodomain parts of the two structures is plotted. The resolution at which the FSC drops below the threshold (0.25, dashed line) is indicated and represents the resolution up to which the two structures share significant signal above the noise level. The two structures were in good agreement up to 16-Å resolution.
(TIF)

S7 Fig Purification of human LAMP1 membrane-distal domain and binding to VLPs. (A) SDS-PAGE analysis shows two duplicate fractions (1,2) from LAMP1 purification in addition to the molecular size marker (M). The theoretical size of the LAMP1 construct used is indicated. The smearing of LAMP1 bands is attributed to heavy N-linked glycosylation. (B) Binding of purified LAMP1 to VLPs was tested at pH 8 and pH 5. Binding at pH 8 was only slightly above the background level whereas significant binding was observed at pH 5. Error bars denote standard deviation. Statistical significance was calculated using unpaired T-test with Welch’s correction. Significance P<0.01 is indicated with **.
(TIF)

S8 Fig Western blot of VLP samples at different pH. A molecular size maker (M) is shown and the sizes of the bands are indicated on the left. The sizes corresponding to GP1 and GP2 are indicated on the right. Notice the absence of GP1 band at pH 3.0 and pH 4.0.
(TIF)

S9 Fig Comparison of LASV spike structure at pH 3 to the crystal structure of GP2 ectodomain in post-fusion conformation. Post-fusion structure of lymphocytic choriomeningitis virus (LCMV) GP2 (PDB:3MKO) is shown as a blue ribbon and was filtered to 17-Å resolution for comparison to the LASV spike structure derived from virus-like particles (VLP) at pH 3. Membranes are shown as schematic representations and labeled.
(TIF)

S10 Fig Fitting of GP1 atomic coordinates into the GP spike EM density. (A,B) GP spike, determined from VLPs at pH 5.0, is shown from the top (A) and from the side (B). Density segments considered in the fitting are numbered (1–4). Insets show the structure of the GP1 (green; PDB:4ZJF). In addition to the ribbon representation, a surface corresponding to GP1 atomic structure filtered to 16-Å resolution is shown. (C) Box-and-whisker-plots indicating the cross-correlation coefficients for 1,000 evenly rotated fits of GP1 atomic coordinates to the segments 1–3 (segment 4 was omitted as it was embedded in the lipid bilayer). The four duplicates (labeled A to D) correspond to the four different chains in the GP1 in the crystal (PDB:4ZJF) and gave similar cross-correlation coefficients as expected. Circles indicate the unique optimized fits. The final best fit with the highest cross-correlation coefficient (0.860) is indicated with an arrow.
(TIF)

S11 Fig Accuracy of tomographic tilt series alignment. Fiducial alignment error mean is plotted for every tilt series in pixels. Most of the tilt series had an alignment error close to 1 pixel.
(TIF)

S12 Fig Contrast transfer function correction of tomographic tilt series. (A) Amplitude is plotted as a function of spatial frequency for one image extracted from a tilt series. The contrast transfer function (CTF, purple) was fitted to the radial power spectrum (cyan) calculated from several tiles extracted from each tilted image. The frequency range used for fitting is indicated. (B) Defocus, estimated from the fitted CTF illustrated in *A*, is plotted for all the tilts in one tilt series, collected at nominal defocus of 3.5 μm (dashed line). Positive values denote underfocus. Error bars represent minimized error between the actual and fitted curve for each tilt, calculated for the range shown in *A*.
(TIF)

S13 Fig Effect of the starting model and resolution evolution in refinement of the glycoprotein spike. (A) The glycoprotein spike structure derived from virus like particles at pH 7 is shown from the side and from the top both before (C1) and after (C3) applying three-fold symmetry. Note that the trimeric nature of the spike is evident even before C3 symmetry is applied (C1). (B) In addition to using an unbiased starting model with cylindrical averaging (C360), no symmetry (C1) or with the correct symmetry (C3), we tested the effect of wrong starting model symmetry (red dashed boxes) on the resulting average. Results are shown after running 12 iterations of refinement without imposing any symmetry and limiting the resolution to 29-Å resolution. The three-fold, trimeric appearance of the spike was retained in all cases. (C) Four different stages of the refinement are shown. Asymmetric, low-resolution starting model (iteration 0) was used to refine the structure first without symmetry (iteration 4). After the three-fold symmetry had become evident, it was applied (iteration 5), and refinement was run to reach the final resolution of 14 Å (iteration 20).
(TIF)

S14 Fig Comparison of two different refinement strategies. (A) In addition to the standard Dynamo ‘push-back’ refinement strategy, GP averages from the virions were calculated using a custom ‘gold-standard’ refinement strategy. The final results from both refinements are shown from the top and from the side. (B) Fourier shell correlation (FSC) is plotted for averages shown in *A*. Both strategies indicated similar nominal resolution; push-back (FSC = 0.5 threshold) 13.6 Å and gold-standard (FSC = 0.143 threshold) 12.3 Å. However, the lower frequencies were stronger in the map calculated using the push-back strategy (shaded area) providing a better quality map.
(TIF)

S1 Text Supplementary methods. Further detail on the refinement of the sub-tomogram averages.
(DOCX)
