# Supplementary figures and images for: Acidic pH-Induced Conformations and LAMP1 Binding of the Lassa Virus Glycoprotein Spike
Source: PLoS Pathog. 2016 Feb 5;12(2):e1005418. doi: 10.1371/journal.ppat.1005418 (PMC4743923; doi:10.1371/journal.ppat.1005418)

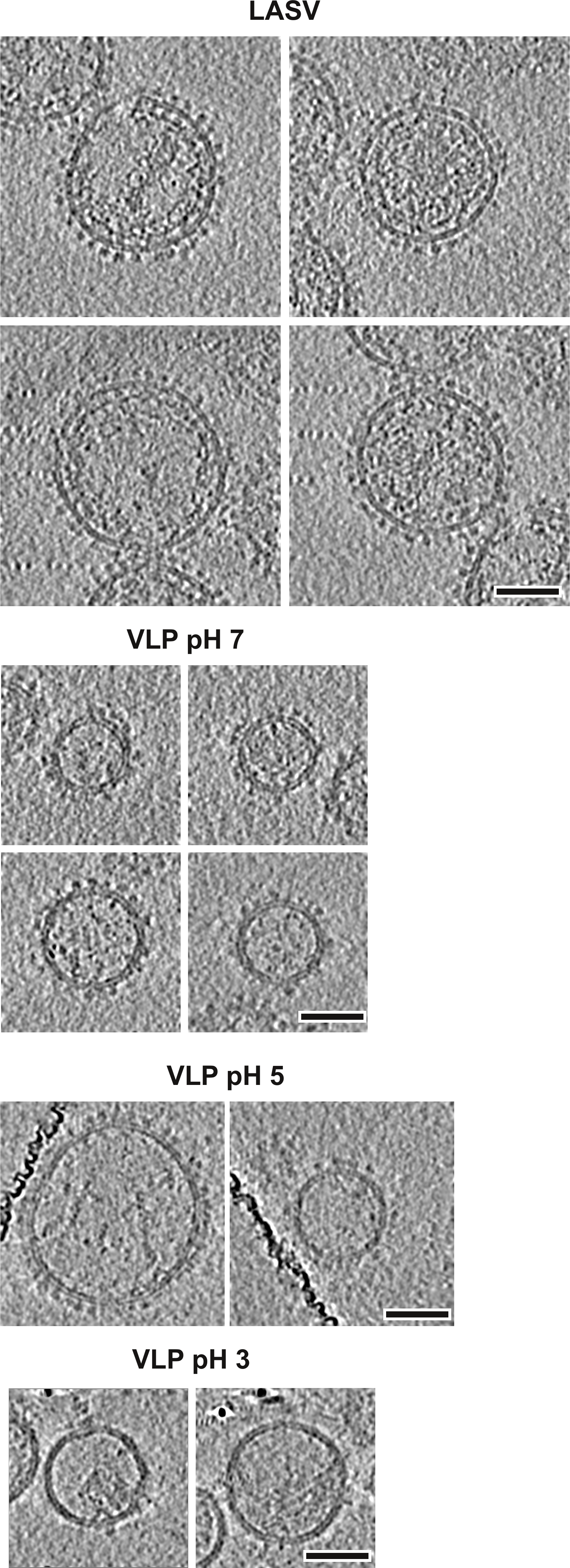

Supplement: S1 Fig — 8-nm thick slices through three-dimensional tomographic reconstructions of LASV and VLPs at different pH low-pass filtered to 60-Å resolution are shown. Scale bars, 50 nm. (TIF) [file ppat.1005418.s001.tif]

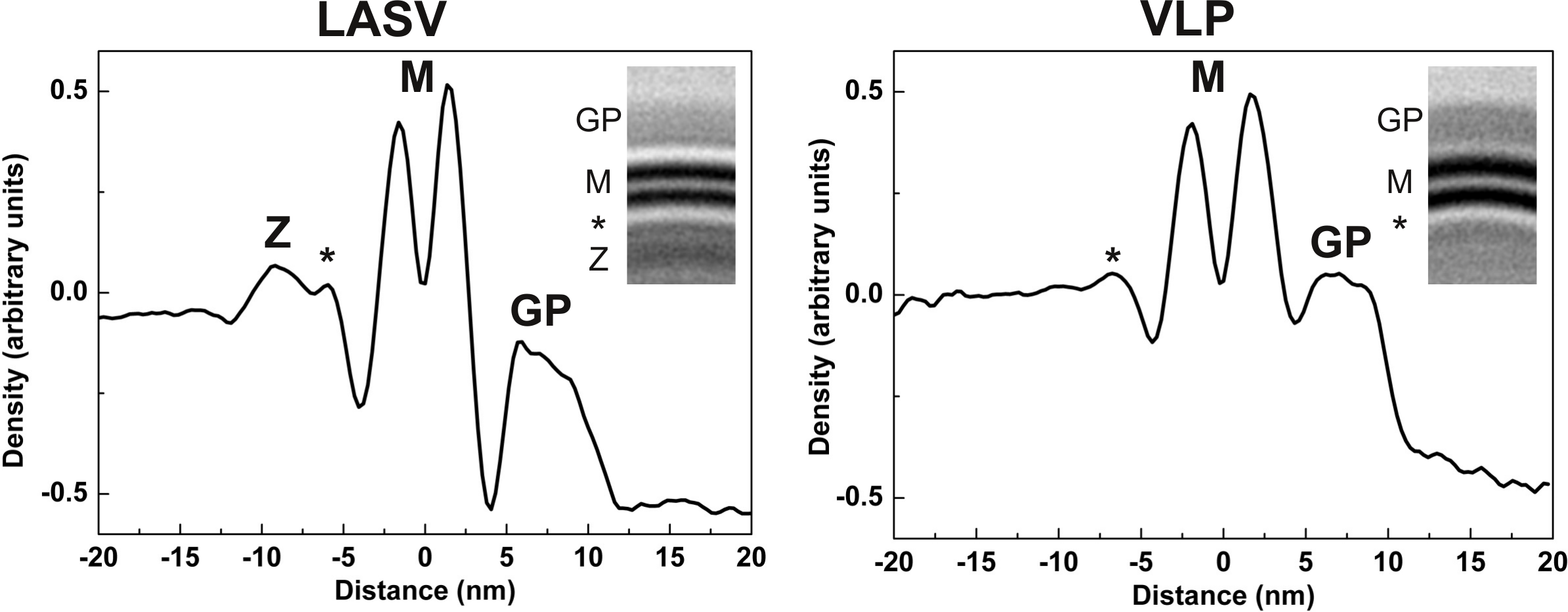

Supplement: S3 Fig — Plots showing the average density distribution in LASV and VLP at pH 7. The insets show a slice through the averaged density, from which the plots were calculated. The glycoprotein layer (GP), membrane (M) and matrix layer (Z) are indicated. The GP intra-viral tails are marked with an asterisk. Notice that the peak assigned to the intra-viral tails is present in both LASV and VLP, but whereas the peak assigned to the Z-layer in present in LASV it is absent in VLP as expected. Both plots have been centered to the middle of the lipid bilayer. (TIF) [file ppat.1005418.s003.tif]

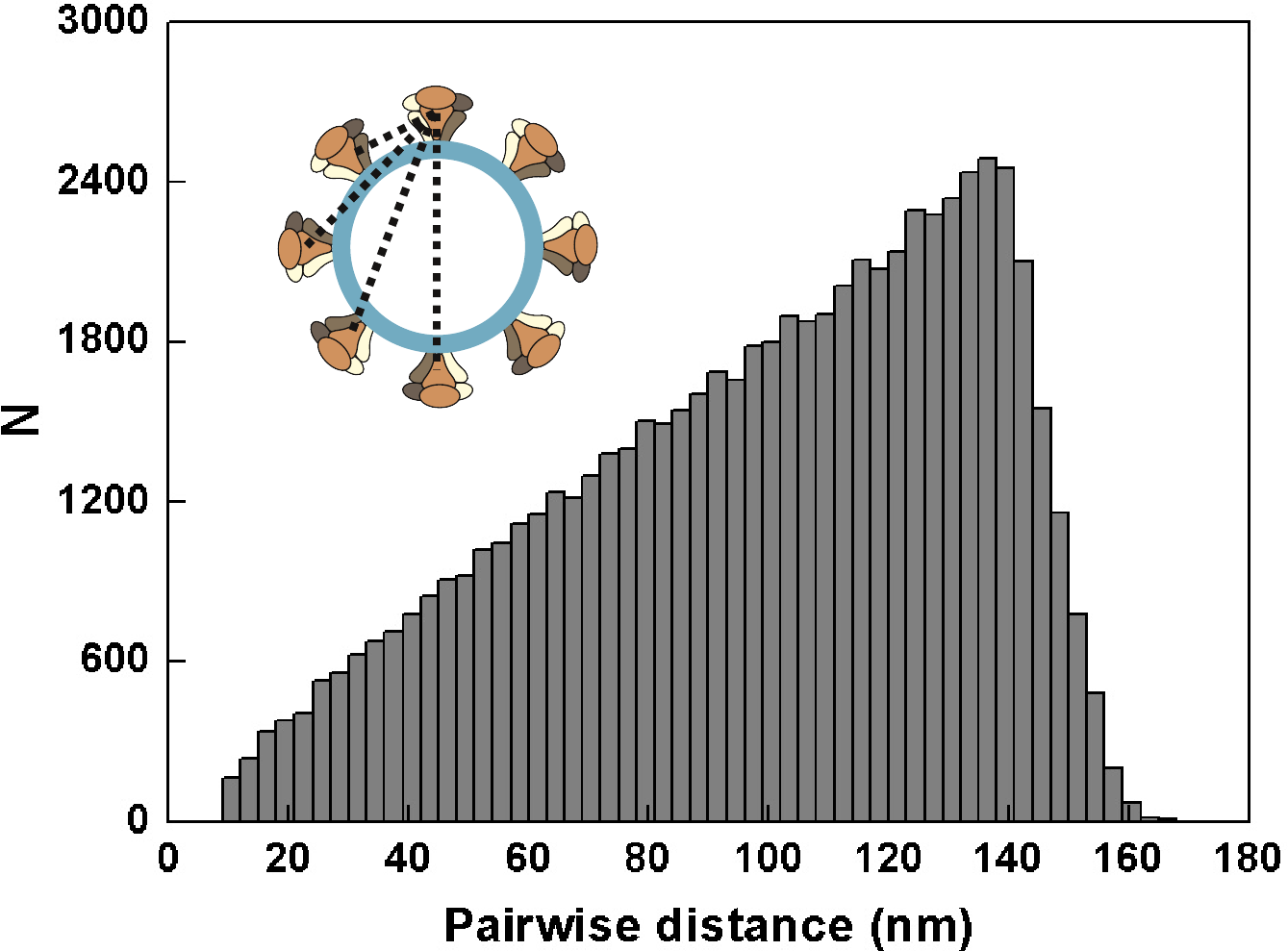

Supplement: S5 Fig — Histogram of pairwise distances of 13,060 spikes found by template-matching on 48 LASV virions is shown. Each bin of the histogram is 3 nm wide. The smallest pairwise distance (~10 nm) reflects the closest spike-to-spike packing corresponding to the size of the spike. The largest pairwise distance (~170 nm) reflects the size of the largest virions in the data set. (TIF) [file ppat.1005418.s005.tif]

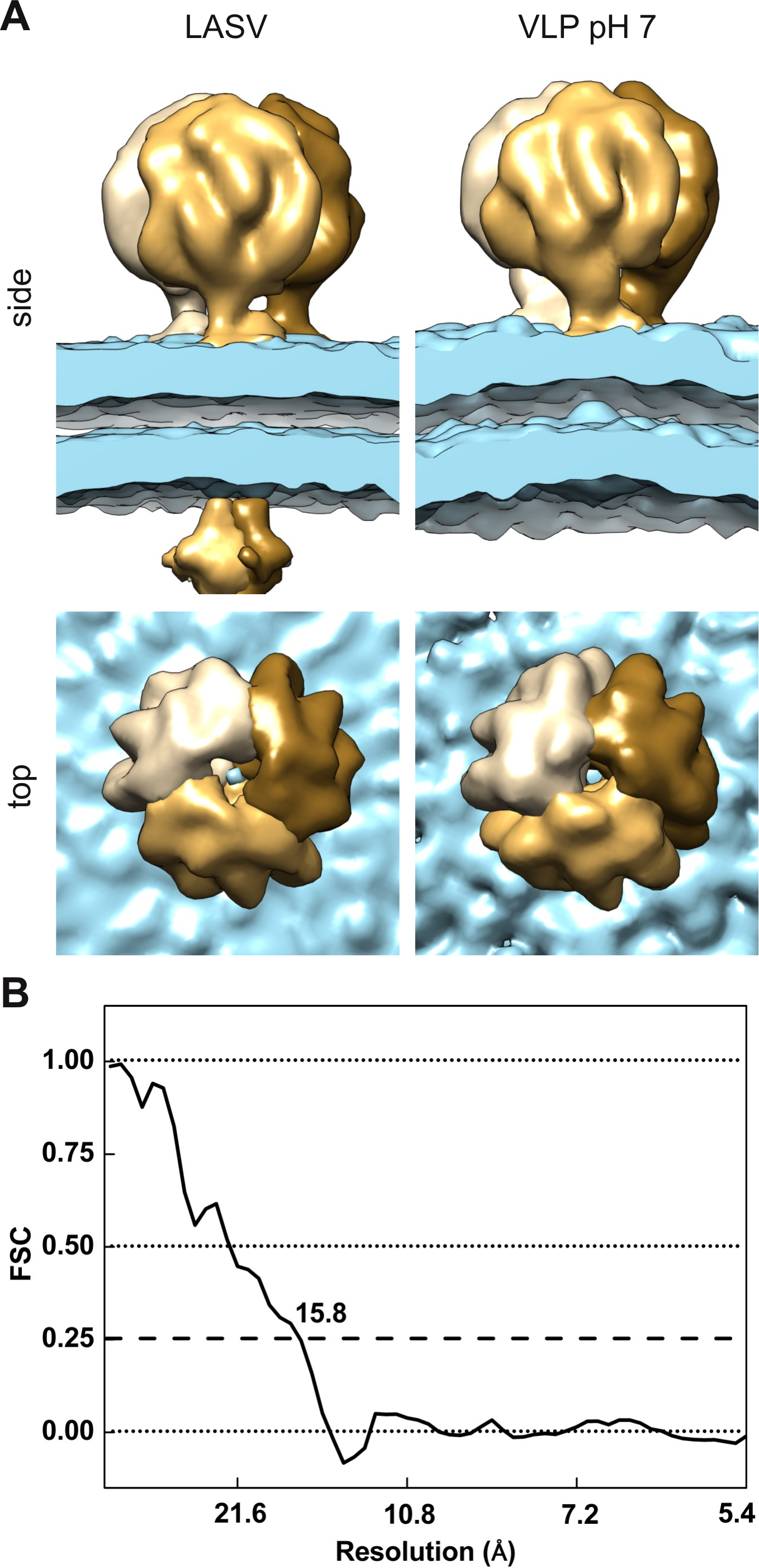

Supplement: S6 Fig — (A) An isosurface representation is shown for the GP structure derived from fixed virions (LASV) and from virus-like particles (VLP pH 7) from the side and from the top. (B) Fourier shell correlation (FSC) calculated between the spike ectodomain parts of the two structures is plotted. The resolution at which the FSC drops below the threshold (0.25, dashed line) is indicated and represents the resolution up to which the two structures share significant signal above the noise level. The two structures were in good agreement up to 16-Å resolution. (TIF) [file ppat.1005418.s006.tif]

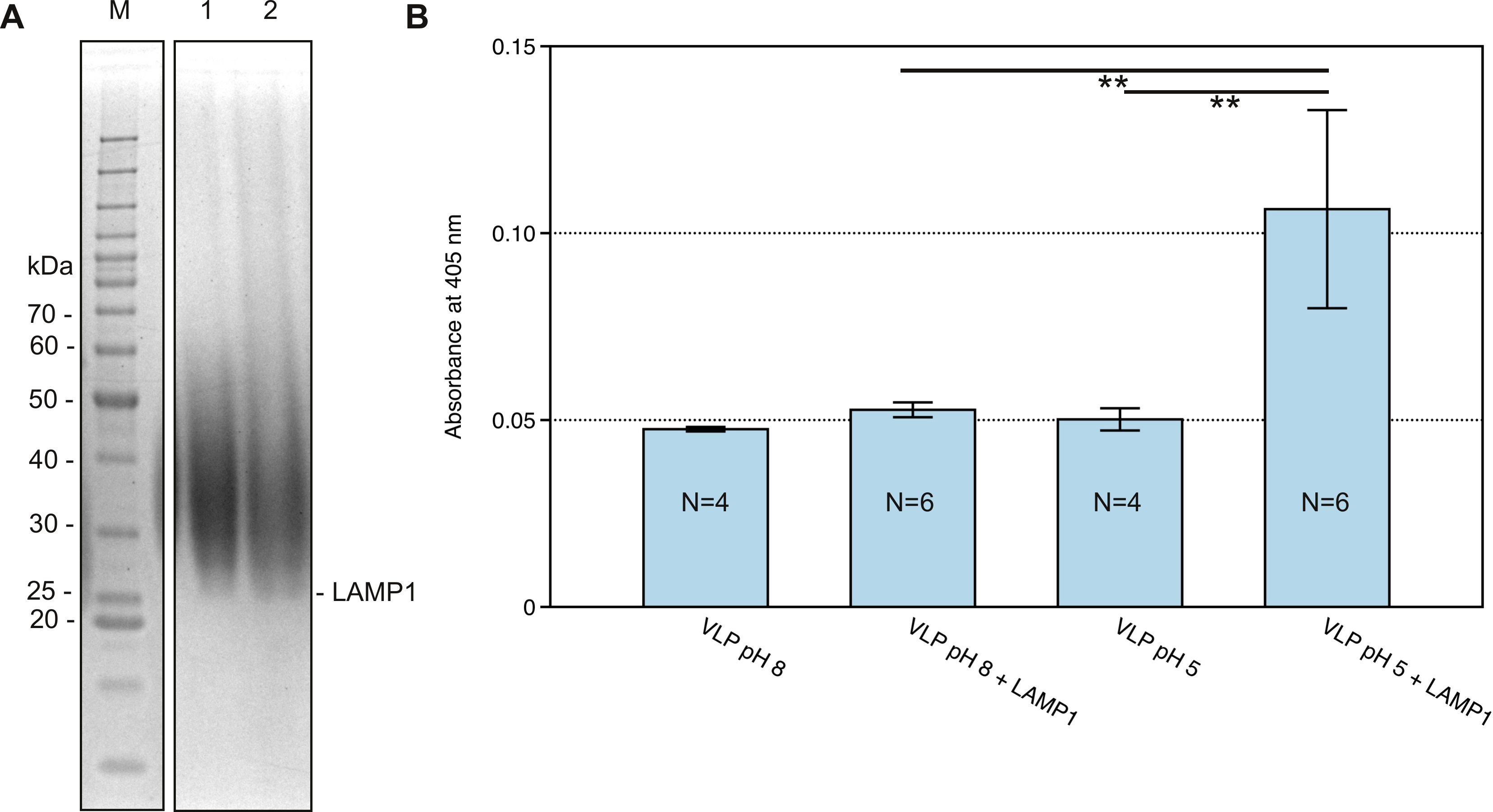

Supplement: S7 Fig — (A) SDS-PAGE analysis shows two duplicate fractions (1,2) from LAMP1 purification in addition to the molecular size marker (M). The theoretical size of the LAMP1 construct used is indicated. The smearing of LAMP1 bands is attributed to heavy N-linked glycosylation. (B) Binding of purified LAMP1 to VLPs was tested at pH 8 and pH 5. Binding at pH 8 was only slightly above the background level whereas significant binding was observed at pH 5. Error bars denote standard deviation. Statistical significance was calculated using unpaired T-test with Welch’s correction. Significance P<0.01 is indicated with **. (TIF) [file ppat.1005418.s007.tif]

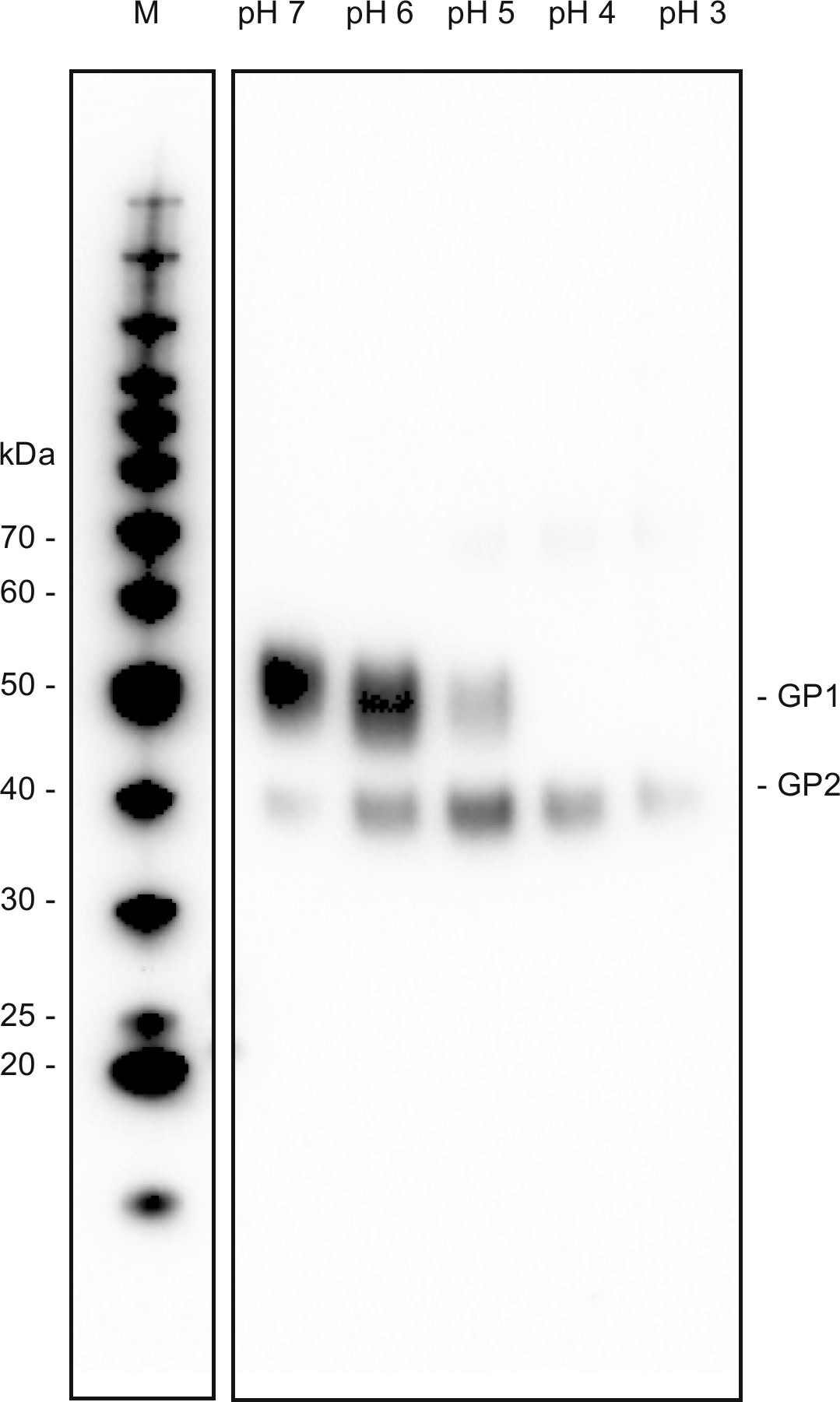

Supplement: S8 Fig — A molecular size maker (M) is shown and the sizes of the bands are indicated on the left. The sizes corresponding to GP1 and GP2 are indicated on the right. Notice the absence of GP1 band at pH 3.0 and pH 4.0. (TIF) [file ppat.1005418.s008.tif]

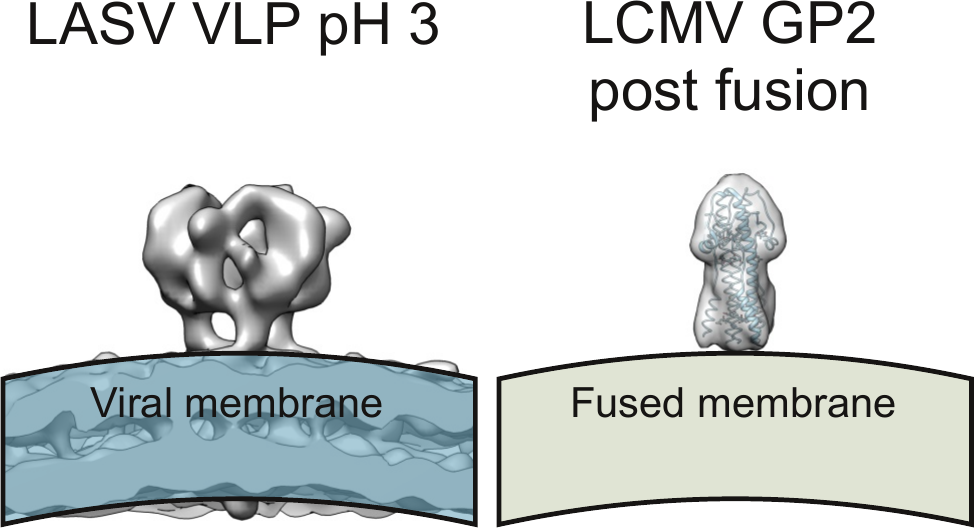

Supplement: S9 Fig — Post-fusion structure of lymphocytic choriomeningitis virus (LCMV) GP2 (PDB:3MKO) is shown as a blue ribbon and was filtered to 17-Å resolution for comparison to the LASV spike structure derived from virus-like particles (VLP) at pH 3. Membranes are shown as schematic representations and labeled. (TIF) [file ppat.1005418.s009.tif]

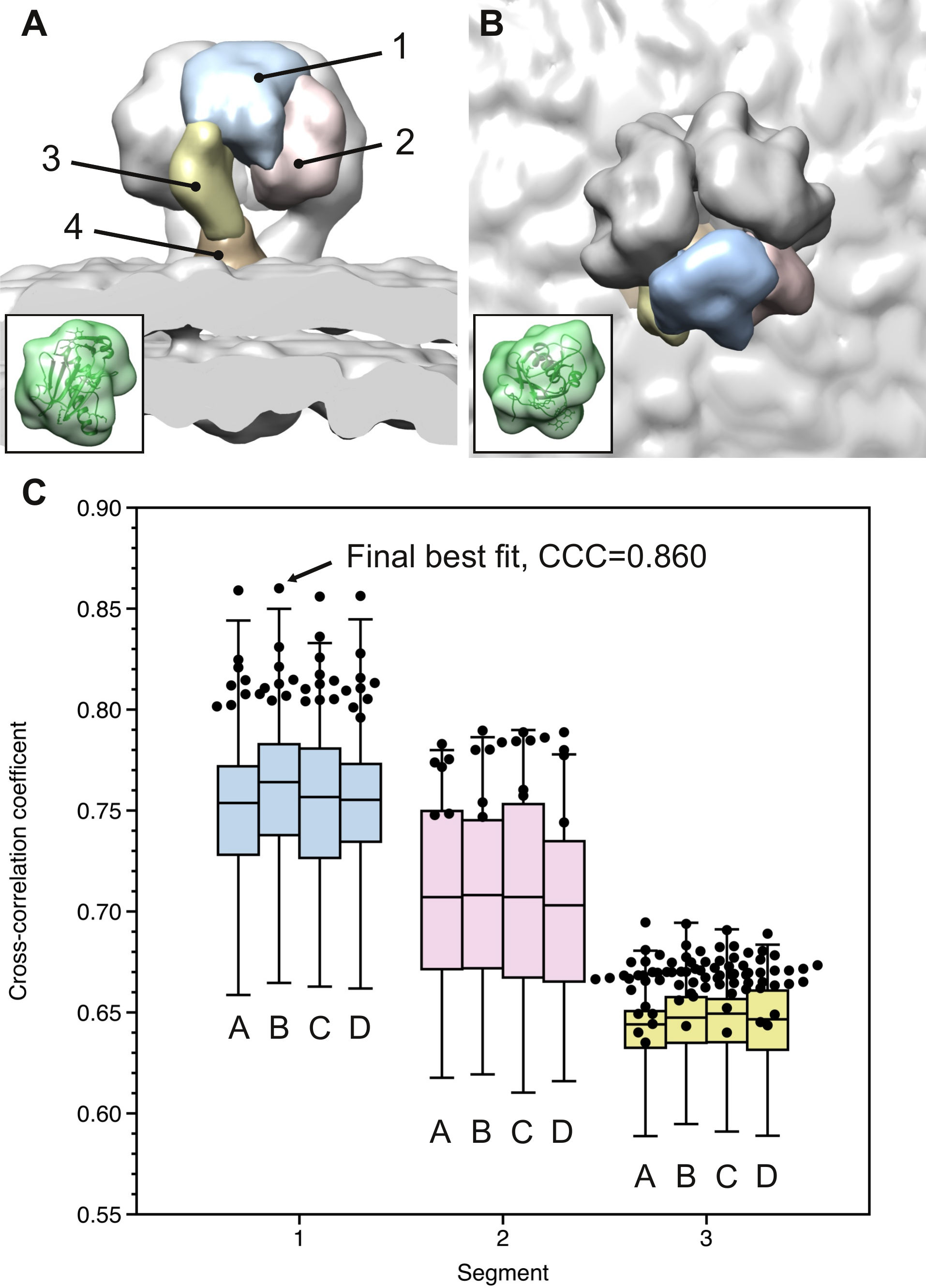

Supplement: S10 Fig — (A,B) GP spike, determined from VLPs at pH 5.0, is shown from the top (A) and from the side (B). Density segments considered in the fitting are numbered (1–4). Insets show the structure of the GP1 (green; PDB:4ZJF). In addition to the ribbon representation, a surface corresponding to GP1 atomic structure filtered to 16-Å resolution is shown. (C) Box-and-whisker-plots indicating the cross-correlation coefficients for 1,000 evenly rotated fits of GP1 atomic coordinates to the segments 1–3 (segment 4 was omitted as it was embedded in the lipid bilayer). The four duplicates (labeled A to D) correspond to the four different chains in the GP1 in the crystal (PDB:4ZJF) and gave similar cross-correlation coefficients as expected. Circles indicate the unique optimized fits. The final best fit with the highest cross-correlation coefficient (0.860) is indicated with an arrow. (TIF) [file ppat.1005418.s010.tif]

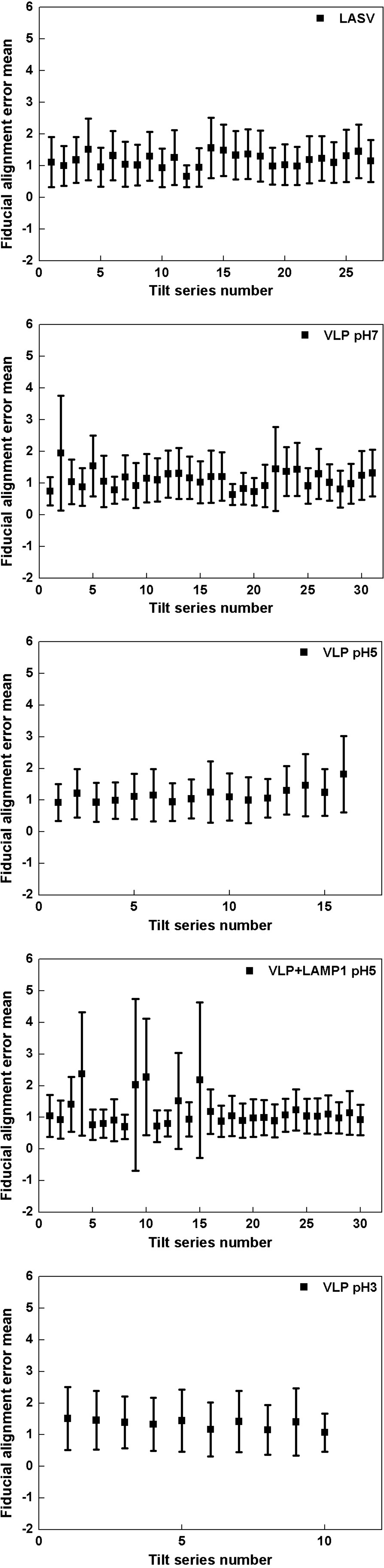

Supplement: S11 Fig — Fiducial alignment error mean is plotted for every tilt series in pixels. Most of the tilt series had an alignment error close to 1 pixel. (TIF) [file ppat.1005418.s011.tif]

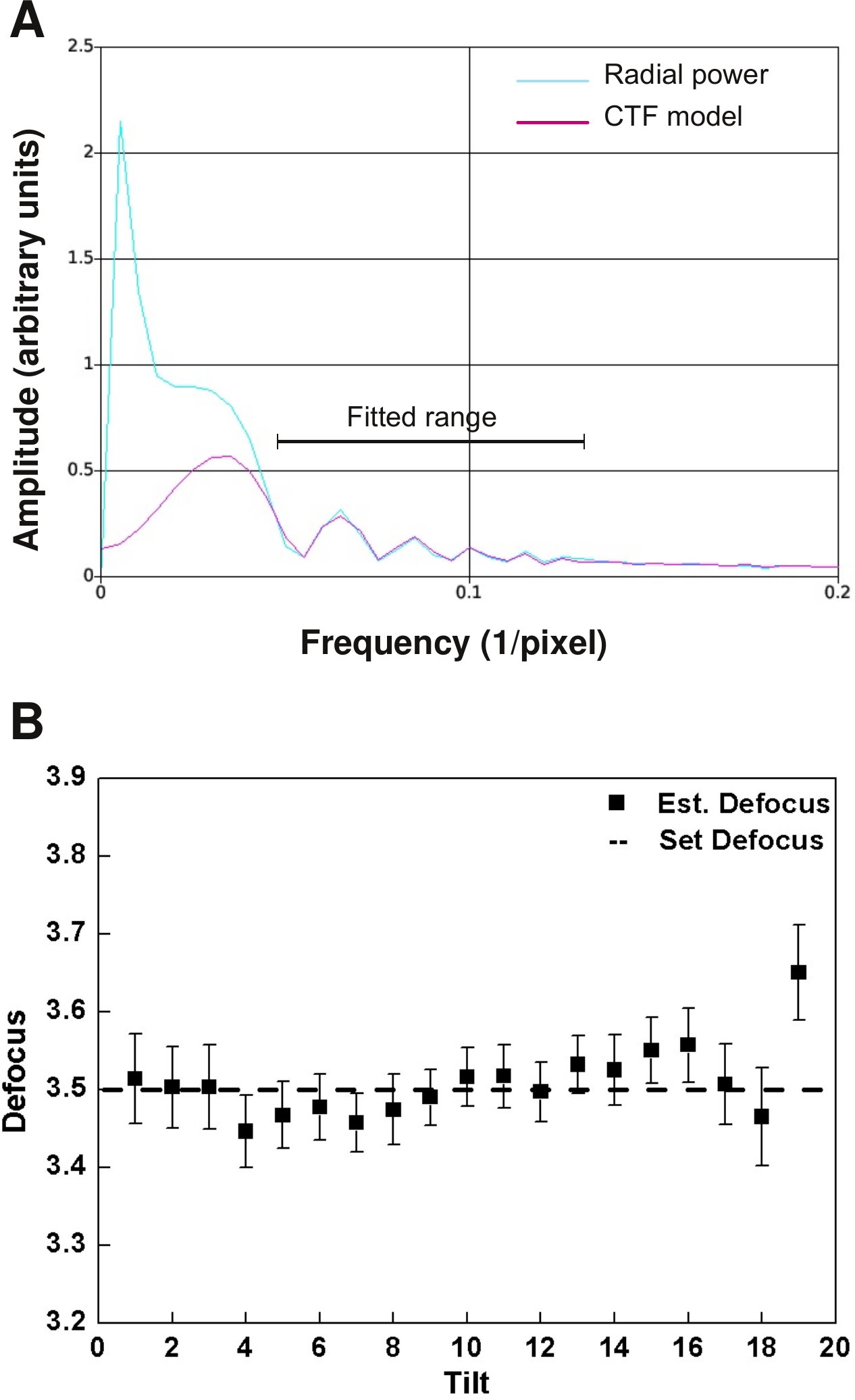

Supplement: S12 Fig — (A) Amplitude is plotted as a function of spatial frequency for one image extracted from a tilt series. The contrast transfer function (CTF, purple) was fitted to the radial power spectrum (cyan) calculated from several tiles extracted from each tilted image. The frequency range used for fitting is indicated. (B) Defocus, estimated from the fitted CTF illustrated in A, is plotted for all the tilts in one tilt series, collected at nominal defocus of 3.5 μm (dashed line). Positive values denote underfocus. Error bars represent minimized error between the actual and fitted curve for each tilt, calculated for the range shown in A. (TIF) [file ppat.1005418.s012.tif]

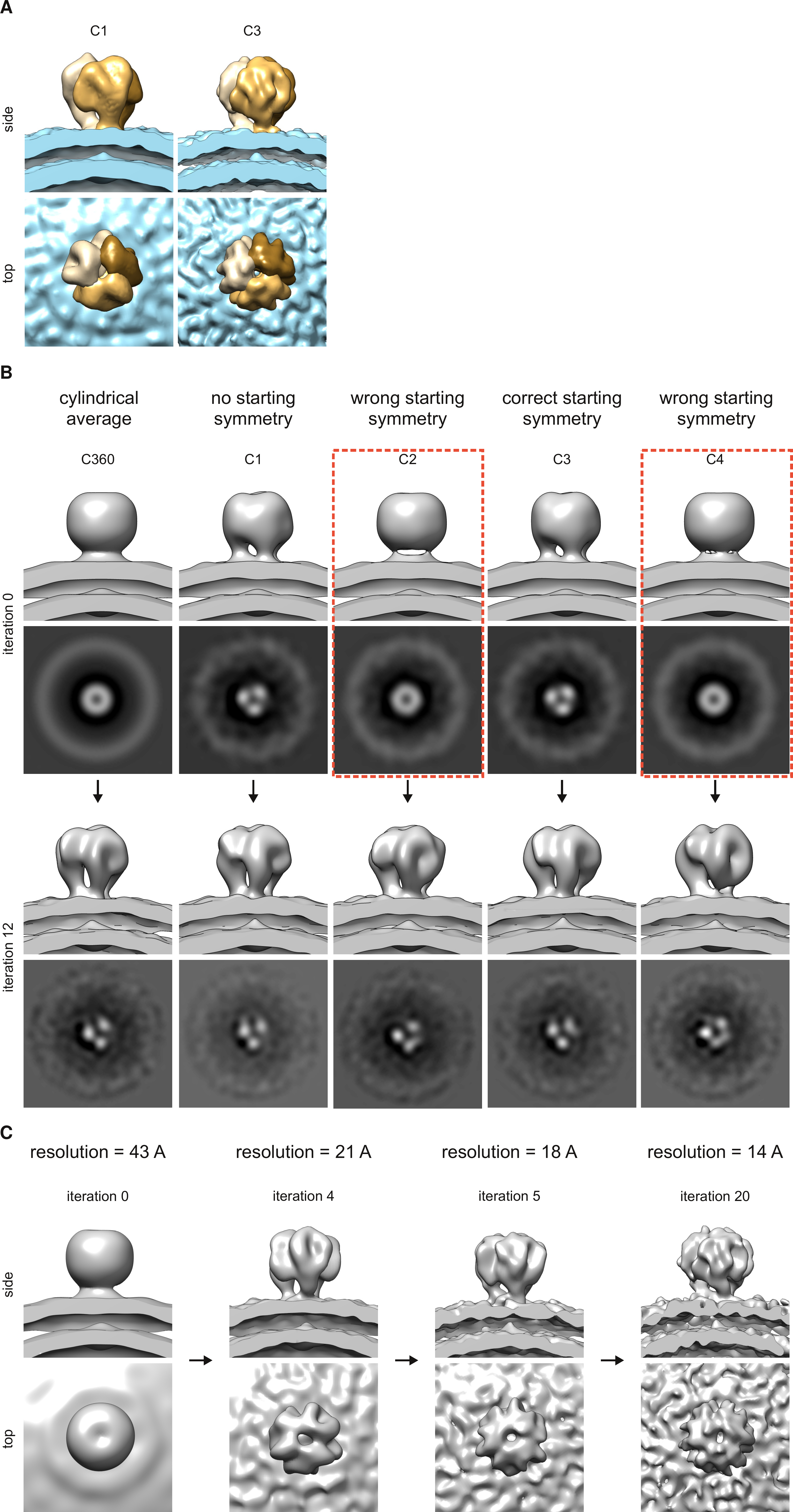

Supplement: S13 Fig — (A) The glycoprotein spike structure derived from virus like particles at pH 7 is shown from the side and from the top both before (C1) and after (C3) applying three-fold symmetry. Note that the trimeric nature of the spike is evident even before C3 symmetry is applied (C1). (B) In addition to using an unbiased starting model with cylindrical averaging (C360), no symmetry (C1) or with the correct symmetry (C3), we tested the effect of wrong starting model symmetry (red dashed boxes) on the resulting average. Results are shown after running 12 iterations of refinement without imposing any symmetry and limiting the resolution to 29-Å resolution. The three-fold, trimeric appearance of the spike was retained in all cases. (C) Four different stages of the refinement are shown. Asymmetric, low-resolution starting model (iteration 0) was used to refine the structure first without symmetry (iteration 4). After the three-fold symmetry had become evident, it was applied (iteration 5), and refinement was run to reach the final resolution of 14 Å (iteration 20). (TIF) [file ppat.1005418.s013.tif]

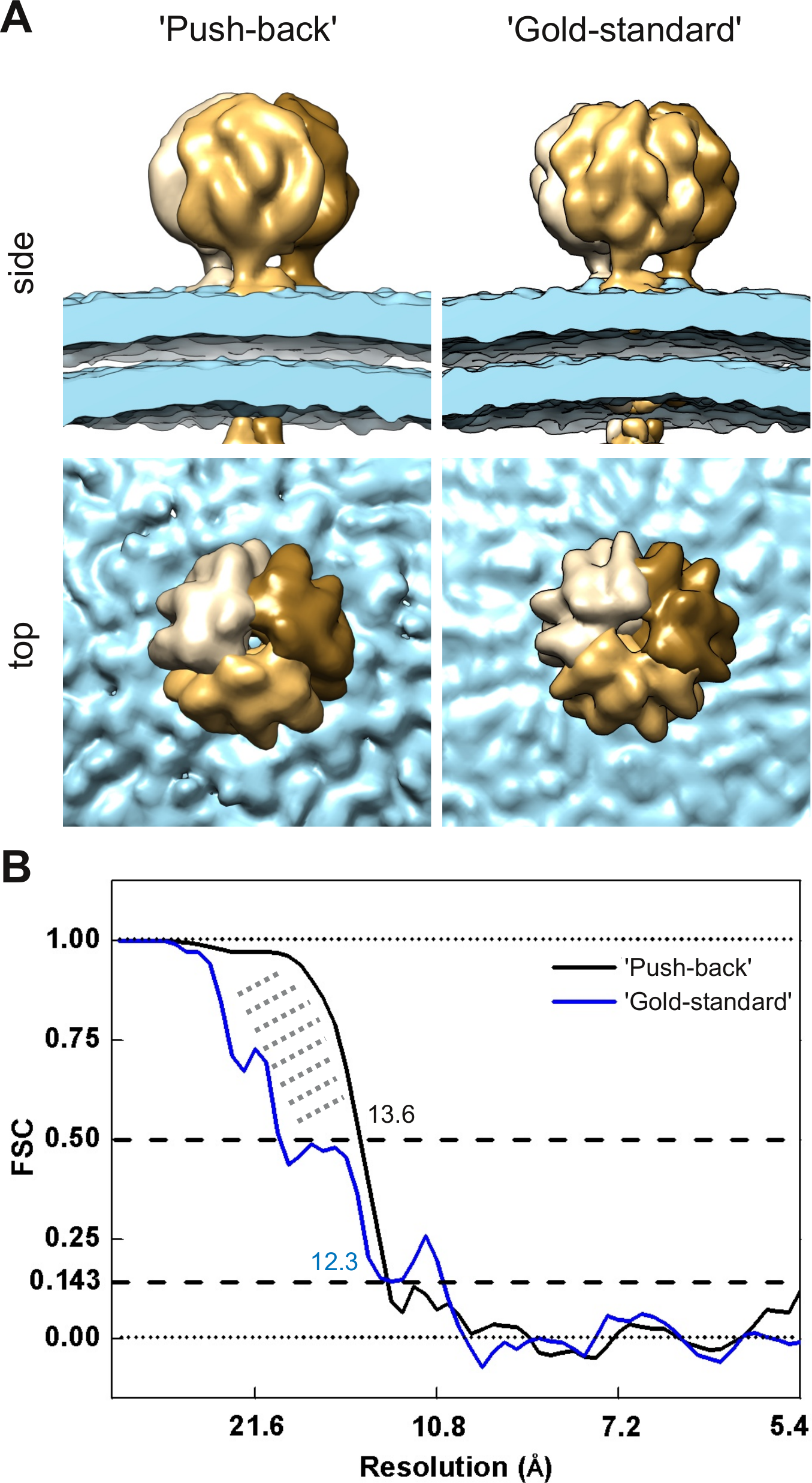

Supplement: S14 Fig — (A) In addition to the standard Dynamo ‘push-back’ refinement strategy, GP averages from the virions were calculated using a custom ‘gold-standard’ refinement strategy. The final results from both refinements are shown from the top and from the side. (B) Fourier shell correlation (FSC) is plotted for averages shown in A. Both strategies indicated similar nominal resolution; push-back (FSC = 0.5 threshold) 13.6 Å and gold-standard (FSC = 0.143 threshold) 12.3 Å. However, the lower frequencies were stronger in the map calculated using the push-back strategy (shaded area) providing a better quality map. (TIF) [file ppat.1005418.s014.tif]
