# Supplementary material for: Acidic pH-Induced Conformations and LAMP1 Binding of the Lassa Virus Glycoprotein Spike
Source: PLoS Pathog. 2016 Feb 5;12(2):e1005418. doi: 10.1371/journal.ppat.1005418 (PMC4743923; doi:10.1371/journal.ppat.1005418)
Supplement: S1 Text — Further detail on the refinement of the sub-tomogram averages. (DOCX) [file ppat.1005418.s015.docx]

Supporting information to:

Acidic pH-induced Conformations and LAMP1 binding of the Lassa Virus Glycoprotein Spike

**Electron Cryotomography Structure of Lassa Virus**

Sai Li^1^, Zhaoyang Sun^1^, Rhys Pryce^1^, Marie-Laure Parsy^1^, Sarah K. Fehling^2^, Katrin Schlie^2^, C. Alistair Siebert^1^, Wolfgang Garten^2^, Thomas A. Bowden^1^, Thomas Strecker^2,*^, Juha T. Huiskonen^1,*^

^1^ Division of Structural Biology, Wellcome Trust Centre for Human Genetics, University of Oxford, Oxford, United Kingdom

^2^ Institute of Virology, Philipps Universität Marburg, Marburg, Germany

* Correspondence should be addressed to T.S. (strecker@staff.uni-marburg.de) or J.T.H (juha@strubi.ox.ac.uk)

# Supplementary Methods

**Refinement of the sub-tomogram averages**

In the first stage of refinement, both the locations and directions of the picked particles were allowed to change to roughly align the spikes. The angle around the spike long axis (azimuth) was kept fixed. The resolution was restricted to 35 Å, and a large spherical mask (radius 63 pixels) and full cylindrical symmetrization were applied. In the second stage, only the azimuth angle was refined. A small mask (radius 20 pixels), encompassing only the spike ectodomain was used, and no symmetry was assumed. After the second stage, three-fold symmetry was evident, especially in the spike base, and was applied in the subsequent iterations (S13A Fig.). In the third stage, the azimuth angle was further refined taking into account three-fold symmetry. In the fourth stage, all six parameters (three location coordinates and three Euler angles) were allowed to change simultaneously.

To prevent over-refinement (correlation of noise instead of signal with the used template structure) during the refinement strategy described above, we adopted the default refinement scheme implemented in Dynamo, which uses adaptive low-pass filtering. After each iteration, the map was first masked and symmetrized using a custom plugin and the resolution of the reconstruction was estimated by FSC. The Fourier shell, where the FSC drops below 0.5, represents the limit of signal in the reconstruction [1]. In the standard Dynamo refinement used here, two shells were subtracted from this value to set a more conservative low-pass limit (‘push-back’ option). Alternatively to the adaptive low-pass filtering with ‘push-back’ option, so-called ‘gold-standard’ refinement is commonly used to prevent over-fitting [2]. There the data is divided into two independent data sets right from the beginning and the low-pass filter parameters are decided based on Fourier shell correlation (FSC=0.143). To further test for possible biases in our refinement strategy, we carried out such gold-standard refinements in Dynamo using a custom plugin implemented for this purpose. These test runs revealed practically the same trimeric, tripodal structure of the spike (S14 Fig.).

# References

1. Rosenthal PB, Henderson R. Optimal determination of particle orientation, absolute hand, and contrast loss in single-particle electron cryomicroscopy. J Mol Biol. 2003;333: 721–745.

2. Scheres SHW, Chen S. Prevention of overfitting in cryo-EM structure determination. Nat Methods. 2012;9: 853–854. doi:10.1038/nmeth.2115
